# Emotion-Driven Moral Evaluation: A Mechanistic Study Based on the Drift-Diffusion Model

**DOI:** 10.3390/brainsci14101005

**Published:** 2024-10-04

**Authors:** Junfei Lin, Xinlu Zhao, Nian Zhao, Tour Liu

**Affiliations:** 1 Key Laboratory of Adolescent Cyberpsychology and Behavior, Ministry of Education of the People’s Republic of China, Wuhan 430079, China; junflin0915@hotmail.com; 2 Key Laboratory of Human Development and Mental Health of Hubei Province, Wuhan 430079, China; 3 Faculty of Psychology, Tianjin Normal University, Tianjin 300387, China; 1810053@bsu.edu.cn (X.Z.); zhaonian_zn@outlook.com (N.Z.); 4 Key Research Base of Humanities and Social Sciences of the Ministry of Education, Academy of Psychology and Behavior, Tianjin Normal University, Tianjin 300387, China; 5 Tianjin Key Laboratory of Student Mental Health and Intelligence Assessment, Tianjin 300387, China

**Keywords:** moral evaluation, drift-diffusion model, moral vocabulary, cognitive mechanism

## Abstract

Background: Moral evaluation is identified as the first stage in the theory of moral judgment, and academics believe that it may align with the social intuitionist model. This study aims to prove that the model’s emotional dominance hypothesis applies to moral evaluation by presenting a computational decision-making model that mathematically formalizes this emotional dominance decision-making process. We also compared different types of valence evaluation tasks to test the emotional priority hypothesis. Methods: We used a convenience sampling method to randomly recruit 30 enrolled college students. The drift-diffusion model was employed to analyze reaction times for words with various emotional and moral valences Additionally, we designed different valence evaluation tasks based on the response relevance hypothesis and evaluated the processing order through reaction time comparisons. Results: The analysis revealed that the emotional mechanism of immoral evaluation differs from moral evaluation. An increase in emotional valence accelerates the speed of evidence accumulation (v) for moral evaluation (*M* = 1.21, 0.2% < 0 < 99.8%) but lowers decision caution (a) in immoral evaluation (*M* = −0.64, 96.1% < 0 < 3.9%). In contrast, moral valence does not have a significant influence on evaluation processes (v, *M* = −0.28, 72.1% < 0 < 27.9%; a, *M* = −0.32, 79.3% < 0 < 20.7%). Furthermore, We found no significant difference in reaction times between moral and immoral words in the emotional evaluation task (*F*(1,29) = 0.55, *p* = 0.464, partial *η*^2^ = 0.02), but a significant difference existed in the moral evaluation task (*F*(1,29) = 17.99, *p* < 0.001, partial *η*^2^ = 0.38), indicating that the tendency of relatively fast immoral evaluation in emotional evaluation tasks may be caused by emotional priority. Conclusions: Our findings support the intuitive model’s emotional dominance hypothesis and introduce a new emotional mechanism into moral evaluation. This study clarifies the distinct emotional processes in moral and immoral evaluations, fills a gap in the research on moral evaluation, and offers insights into human decision-making in moral contexts.

## 1. Introduction

Morality plays a crucial role in social life. Currently, both the business and healthcare sectors face numerous moral challenges, which vary significantly across different cultural contexts [1]. This underscores the necessity of a deep understanding of moral cognition in varying cultural backgrounds. Specifically, morality refers to the principles that guide individuals in distinguishing right from wrong and making decisions that uphold social norms. In order to maintain coherence and avoid actions that violate collective moral principles, which might hinder access to key social and psychological benefits, individuals must evaluate moral situations and respond appropriately [2]. In recent years, an increasing number of studies have found that individuals are significantly influenced by emotions when assessing moral situations; for instance, individuals with low empathy tend to disregard moral norms when they receive negative emotional feedback [3]. To explore the impact of emotions on personal decision-making, the valence-based approach has emerged as an effective method for elucidating the relationship between emotions and decisions. This approach has been widely applied in studies examining impulsive emotions and consumer behavior [4] as well as in emotional theory [5]. Furthermore, the valence-based approach has been extensively utilized in the field of moral judgment. For example, participants experiencing feelings of anger are more likely to make strong moral judgments, showing reduced tolerance for violations [6] or expressing stronger punitive intentions [7]. Within this process, moral evaluation refers to the initial stage in which individuals quickly assess the morality of a given situation [8]. Research has shown that moral evaluation occurs early in the moral judgment process [9].

## 2. Review of the Literature and the Theoretical Background of This Study

### 2.1. The Social Intuitionist Model

The process of moral evaluation may be explained by the social intuitionist model [8]. As an important model used to explain moral judgment in the field of moral psychology, it asserts that moral judgment is driven by immediate moral intuitions, with subsequent slower moral reasoning occurring only when necessary [10]. This statement contains two claims about the intuitive nature of moral judgment. One claim concerns the processing order, asserting that emotion takes precedence in processing, with reasoned thought serving as a post-justification. This view is supported by previous research, which found a phenomenon known as “moral dumbfounding”, where individuals sometimes perceive certain behaviors as morally wrong yet are unable to provide a justification for this belief [11,12,13]. The second claim posits that emotions directly dominate moral judgment. In a hypnotic study [14], induced participants to associate certain words with disgust and found that certain words aggravated moral condemnation associated with immoral descriptions [15] reported similar results, with disgust induced by fart spraying or disgusting videos leading to stricter moral judgment. From these two hypotheses of the model, moral evaluation seems to be an emotional moral judgment process.

However, the extension of the social intuitionist model to moral evaluation needs to be tested. The hypothesis of emotion priority in the social intuitionist model is primarily based on the phenomenon of “moral dumbfounding”, which is problematic when applied to moral evaluation. First, “moral dumbfounding” may not equate to moral evaluation. In fact, behavioral evidence indicates that this evaluation concludes within 1600 ms [16]; electrophysiological data reveal that the moral evaluation process occurs between 300 and 600 ms [17,18]. However, in the publicly available database of “moral dumbfounding” studies [13], the average reaction time for moral judgment is longer than the 1600 ms defined for moral evaluation [16]. This suggests that the “moral dumbfounding” phenomenon may entail not just moral evaluation but also norm judgment [8]. Second, the inference of processing order from this phenomenon may be biased. Studies have shown significant variations in the incidence rates of “moral dumbfounding” across various scenarios [13]. This is because “moral dumbfounding” is verified by descriptive analyses of the incidence rate, rather than by a direct index to reflect processing [12,13,19], which results in a large influence of social expectations on the experimental results [20,21], and this unstable result makes it difficult to generalize the processing order to moral evaluation.

The hypothesis that emotions play a dominant role is also problematic when extended to moral evaluation. Initially, this hypothesis was based on research indicating that irrelevant disgust aggravates immoral judgment [14,15]. The issue is that these studies have strong habits of studying immoral judgment, but moral evaluation involves both immoral and moral conditions. Thus, the impact of emotions on immoral judgment should not be generalized to moral judgment. Furthermore, a meta-analysis revealed that disgust had an amplifying impact on moral judgment, potentially overstating the effect of emotion factor in moral judgment [22]. According to the study, induced disgust has a small impact on moral judgment (*d* = 0.11), and once publication bias is corrected, the effect vanishes, suggesting that the effect of emotions on moral judgment is unstable. Lastly, studies that induce irrelevant stimuli may reveal which factors dominate moral judgment, but it is hard to investigate how these factors affect the moral judgment process since these irrelevant stimuli themselves are not engaged in moral judgment process.

In summary, it is unclear whether the social intuitionist model extends to moral evaluation. Here, we aim to fill this gap. First, we introduce the response relevance hypothesis to validate the emotional priority hypothesis, followed by the drift-diffusion model to test the emotional dominance hypothesis. The methods section details our experimental design and analysis procedures. Finally, we present results regarding the processing order of emotion and reason. We also provide behavioral data that support the emotional dominance hypothesis and outcomes from the drift-diffusion model. At the end of the paper, we discuss the study’s conclusions and limitations.

### 2.2. The Response Relevance Hypothesis

Moral vocabulary and the response relevance hypothesis it follows can be used to distinguish the factors of emotion and reason in moral evaluation, thereby examining the emotional priority hypothesis. Specifically, moral vocabulary has two attributes: moral valence, which carries moral information, and emotional valence, which carries emotional information [23]. These attributes can be applied in valence evaluation tasks to evaluate positive and negative, as well as morally right and wrong, corresponding to emotional and reasoning processes in moral evaluation. At the same time, moral vocabulary follows the response relevance hypothesis in valence evaluation tasks [24]. The hypothesis demonstrates that negative words (e.g., snake) elicit slower lexical decisions but faster valence judgments compared to positive words (e.g., puppy). This effect is attributed to the negative bias [25], where negative words attract attentional resources more quickly due to their negative content, requiring more time to disengage from response-irrelevant tasks to respond appropriately, such as judging true and false words. However, this enables quicker reactions in response-relevant tasks, like evaluating positive and negative words. Similarly, we speculate that the slower response to immoral words in the moral evaluation task may be due to their engagement in response-irrelevant tasks, necessitating additional time for individuals to separate their attention from negative emotions to accurately respond to morally right or wrong task [26,27,28]. Following this logic, if immoral words (with negative valence, e.g., betrayal) yield quicker responses than moral words (e.g., loyalty) in emotional evaluation tasks, this indicates that immoral words with negative emotions capture attentional resources more rapidly. This strengthens the evidence that the slower response to immoral words is a result of prioritized emotional processing, thus supporting the applicability of the emotional priority hypothesis in the social intuitionist model for moral evaluation.

### 2.3. The Drift-Diffusion Model

**Figure 1:**
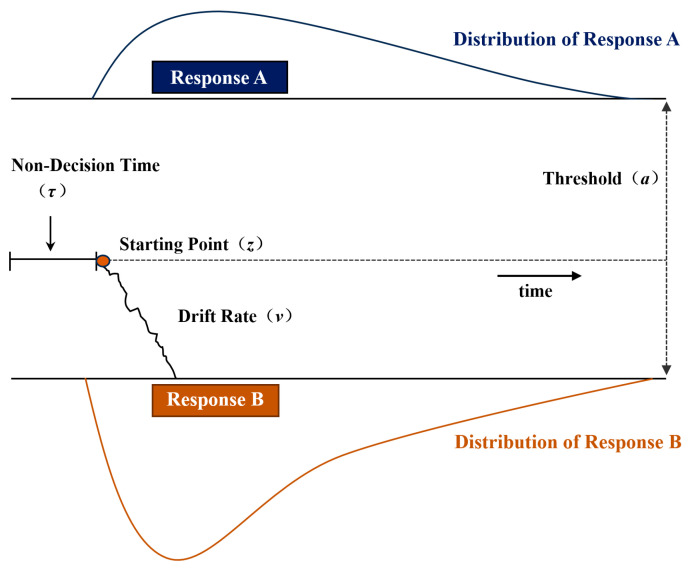
The drift-diffusion model.

To test the emotional dominance hypothesis, the drift-diffusion model (DDM) combining the functional separation method [23] is used to capture the cognitive mechanism of emotion and reason in moral evaluation. This model has been applied to various moral evaluation tasks [29,30,31,32] and has received extensive attention for its ability to fit choices and reaction times to quantify the psychological mechanisms of decision-making [33,34,35,36]. It provides a mathematical framework to explain the cognitive mechanisms of fast binary decision-making tasks, viewing binary decisions as a continuous process fluctuating between two potential options. In the model, the decision-making process begins at a starting point situated between the boundaries with two decision responses. As time progresses, individuals gather and accumulate evidence from stimuli, and a decision is made when the cumulative evidence reaches either the upper or lower threshold (Figure 1). In the experiment, each trial had a process of evidence accumulation, and each process of evidence accumulation was recorded after reaching the threshold. Thus, multiple trials formed a distribution of reaction times.

The parameters of the drift-diffusion model quantify various psychological components of the decision-making process, such as the speed of evidence accumulation, degree of decision caution, prior bias, and non-decision time, allowing for inferences about potential cognitive mechanisms [37]. The formula is provided below [38]:

$$f\left(t|z,a,\nu \right)=\frac{\pi }{a^{2}}\cdot \exp\left[\left(a-z\right)\nu \right]\sum_{n=1}^{\infty }\;n\cdot sin\left\lfloor \frac{\pi \left(a-z\right)n}{a}\right\rfloor \cdot \exp\left[-0.5\cdot \left(\nu^{2}+\frac{\pi^{2}n^{2}}{a^{2}}\right)\cdot t\right]$$

In this model, decision-making is driven by a combination of systematic evidence accumulation and random fluctuations. The drift rate (*v*) measures the average speed at which an upper threshold is approached, reflecting perceptual sensitivity or task difficulty. This implies that individuals who are more sensitive to a choice, or who face simpler tasks, will accumulate evidence more rapidly. The upper threshold (*a*) is the distance between the two decision boundaries, indicating when a decision is finalized. This parameter indicates the degree of caution in decision-making, with larger thresholds requiring more evidence and time to make decisions. The starting point (*z*), also known as the prior bias, indicates the initial amount of information needed before making a choice. If the starting point is closer to one of the responses, it suggests an initial bias towards that response before making a decision. The non-decision time (*τ*), calculated as the response time (*rt*) minus the decision time (*t*), accounts for the time spent on information encoding and motor responses [39]. Additionally, we employed a functional separation method [23], which controls one component of the stimulus material (e.g., emotional valence) while varying another (e.g., moral valence), to examine the distinct mechanisms of moral evaluation.

Here, we applied the drift-diffusion model (DDM) to the binary evaluation of moral vocabulary, analyzing reaction time distributions across different lexical valences to explore the decision-making process in moral evaluation. The drift rate (*v*) quantifies the strength of evaluative evidence during the processing of moral stimuli, whereas the upper threshold (*a*) indicates the amount of evidence required for making an evaluation. Furthermore, the starting point (*z*) quantifies the prior bias toward moral or immoral evaluation before receiving any stimulus. These parameters help us quantify the process of moral evaluation under the effect of different moral and emotional valence by combining the functional separation method [23]. Thus, we can investigate how emotional and rational factors influence the moral evaluation process to test the emotional dominance hypothesis.

In summary, our investigation of whether moral evaluation is consistent with the social intuitionist model is motivated by two lines of research. First, we designed valence evaluation tasks based on the response relevance hypothesis, using response time as a metric to infer the processing order of moral evaluation. Second, we used computational models to explore the effects of emotion and reason on moral and immoral evaluations and to quantify the underlying psychological mechanisms.

## 3. Methods

### 3.1. Participants

Using G*Power 3.1 for sample size calculations [40], the analysis indicated that at least 26 participants were required to achieve an 85% statistical power for a within-subjects repeated measures ANOVA with a significance level of *α* = 0.05 and a medium effect size (*f* = 0.25). In this study, a total of 30 college students from a university in Tianjin, China, were recruited (4 males, 26 females; *M* age = 19.8, *SD* = 1.14, range = 19–24). All participants were recruited online. We employed convenience sampling by posting recruitment information on social media. Participants were screened based on native Chinese language proficiency. All participants were undergraduate students, and they received RMB 20 as compensation upon completion of the experiment. No participants were excluded from the analysis. All were native Chinese speakers with normal vision and were right-handed. This study received ethical approval, and all participants provided written informed consent.

### 3.2. Design

This research conducted a 2 (task type: emotional evaluation vs. moral evaluation) × 2 (word type: moral vs. immoral) within-subjects experiment. The emotional evaluation task was designed to elicit the emotional components involved in moral evaluation and to compare with the reason and emotion components activated in the moral evaluation task. This study hypothesized that immoral words would elicit slower reaction times than moral words in the moral evaluation task but faster reaction times in the emotional evaluation task.

### 3.3. Materials

Two-character Chinese words were selected from the extensive moral literature. Next, a moral word inventory was developed by assessing 230 two-character Chinese words using a dual-dimensional questionnaire. The Likert scale and the instructional settings employed in this questionnaire were based on a widely cited study in Chinese vocabulary research [41]. A total of 32 undergraduates evaluated the moral valence (1 = extremely immoral, 5 = neutral, 9 = extremely moral) and emotional valence (1 = extremely negative, 5 = neutral, 9 = extremely positive) on a 1–9 scale (excluding one subject with an answer time greater than two standard deviations, the final questionnaire score included 31 subjects). The questionnaire demonstrated strong reliability, with a Cronbach’s alpha of 0.943 for the emotional dimension and 0.951 for the moral dimension. Words scoring above 2 in either dimension were selected, resulting in 96 two-character Chinese words for experimental material (48 moral words, 48 immoral words).

To ensure that moral and immoral words differed only in their nature, without significant differences in emotional or moral intensity, we transformed the emotional and moral valence scores of both moral and immoral words based on their distance from the neutral midpoint (5). For scores greater than 5, we subtracted 5 to reflect the emotional or moral valence intensity of moral words. For scores lower than 5, we subtracted 5 and took the absolute value to represent the emotional or moral valence intensity of immoral words. This transformation standardizes the deviation from neutrality, allowing for a direct comparison of intensity across both moral and emotional dimensions. An independent *t*-test was conducted on moral and immoral words. The results revealed no significant differences in moral intensity, *t*(94) = 1.01, *p* = 0.317, Cohen’s *d* = 0.229, emotional intensity, *t*(94) = 1.15, *p* = 0.254, Cohen’s *d* = 0.234, strokes, *t*(94) = 0.89, *p* = 0.375, Cohen’s *d* = 0.182, or frequency, *t*(94) = −0.50, *p* = 0.616, Cohen’s *d* = −0.103, between moral and immoral words. The words (see Appendix A Table A1) are suitable for experimental material.

In order to study the effect of different factors by applying the functional separation method, we grouped the moral vocabulary according to emotional and moral valence. We first categorized the 96 moral words into immoral and moral groups, with 48 words in each group. Then, we divided the moral valence scores into three categories (high, medium, low) based on an equal distribution across the valence range, resulting in six categories based on emotional and moral valence (2 × 3). Since no words were classified as low-moral and high-emotional, or high-moral and low-emotional, and words with low emotional and low moral scores (neutral words) were excluded from the materials; the process produced 12 distinct groups of 8 words each, organized by combinations of different valence levels (Table 1).

An independent *t*-test revealed no significant differences in emotional intensity, moral intensity, word frequency, or strokes between the moral and immoral groups within the same emotional and moral categories (*p*s > 0.066) (see Appendix A Table A1).

### 3.4. Procedure

**Figure 2:**
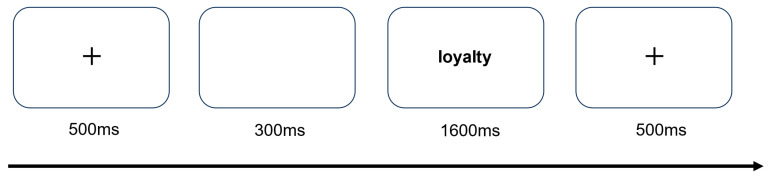
Experimental procedure.

The laboratory environment was quiet and the lighting relatively moderate. Participants read the experimental instructions and began the formal experiment after completing 20 practice trials with an accuracy rate of at least 85%. At the start of each trial, a fixation cross appeared for 500 ms at the center of the screen, followed by a 300 ms blank screen, and then a moral vocabulary stimulus was presented. In the emotional evaluation task, participants evaluated whether the vocabulary was a positive or negative word by pressing the “F” or “J” key. In the moral evaluation task, participants evaluated whether the vocabulary was a moral or immoral word by pressing the “F” or “J” key. Once participants completed the key press or the response time exceeded 1600 ms, they proceeded to the next trial. Each task consisted of 192 trials, with each word presented twice in random order, and participants took a break after every 96 trials. Upon completion of all trials for one task, participants proceeded to the next task. Both key presses and task order were counterbalanced. The experimental procedure is shown in Figure 2.

### 3.5. Trial Selection

This study focused on analyzing reaction time data for different moral evaluations. Therefore, only the accuracy analysis included all trials (11,520 trials across two tasks, with an average of 384 trials per participant). For other analyses, since accuracy was not the primary focus and there was no significant difference in accuracy across different types of moral evaluations, incorrect response time data were excluded in subsequent analyses (348 trials, 6.04% of all moral evaluation trials; 357 trials, 6.20% of all emotional evaluation trials). Additionally, trials that exceeded the evaluation response time limit were excluded (26 trials, 0.45% of all moral evaluation trials; 28 trials, 0.49% of all emotional evaluation trials) [16]. In total, excluding accuracy, we analyzed 5386 moral evaluation trials (per participant *M* = 179.53, *SD* = 6.58, range: [162,191], 93.51% of all moral evaluation trials) and 5375 emotional evaluation trials (per participant *M* = 179, *SD* = 9.09, range: [154,192], 93.31% of all emotional evaluation trials).

### 3.6. Analysis

First, to investigate the order of emotional and reasoning processing in moral evaluation, we analyzed both accuracy and reaction times for moral and emotional evaluation tasks. Second, we explored the effects of emotion and reason factors on moral evaluation through pairwise comparisons of reaction times across various valences. Reaction times for the 12 groups of moral vocabulary were analyzed using paired *t*-tests, with the false discovery rate (FDR) applied to adjust for multiple comparisons [42]. Lastly, to quantify the underlying mechanisms of moral evaluation, we used a drift-diffusion model to fit the distribution of reaction times for the moral evaluation task across different valences.

## 4. Results

### 4.1. The Processing Order of Emotion and Reason

**Figure 3:**
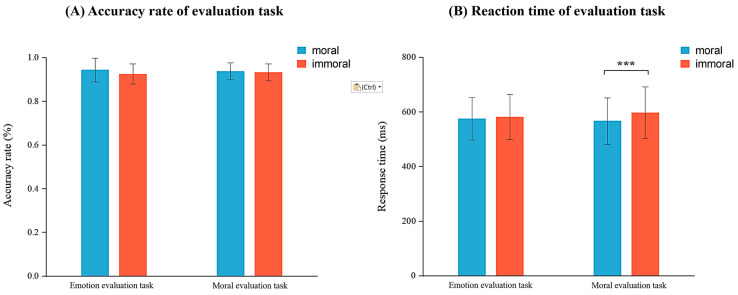
Results of (**A**) accuracy rate and (**B**) reaction time. Note. Error bars indicate the standard deviation. *** *p* < 0.001.

Accuracy rates and reaction times were analyzed using a 2 (task type: emotional evaluation vs. moral evaluation) × 2 (word type: moral vs. immoral) repeated measures ANOVA in emotional and moral evaluation tasks (see Figure 3A). The results of the accuracy rate revealed no significant main effects of task type, *F*(1,29) = 0.09, *p* = 0.766, partial *η*^2^ < 0.01 and of word type, *F*(1,29) = 3.87, *p* = 0.059, partial *η*^2^ = 0.12, or a significant task × word interaction, *F*(1,29) = 3.476, *p* = 0.072, partial *η*^2^ = 0.11. These results suggest that neither task type nor word type significantly influences the accuracy rates in valence evaluation tasks.

Reaction time analysis (see Figure 3B) revealed no significant main effect of task type, *F*(1,29) = 0.30, *p* = 0.587, partial *η*^2^ = 0.01, but showed a significant main effect of word type, *F*(1,29) = 6.83, *p* = 0.014, partial *η*^2^ = 0.20, and a significant task × word interaction, *F*(1,29) = 12.41, *p* = 0.001, partial *η*^2^ = 0.30. Further analysis of simple effects revealed that in the moral evaluation task, individuals responded significantly faster to moral words (*M* = 568.24, *SD* = 86.25) than to immoral words (*M* = 598.00, *SD* = 93.68), *F*(1,29) = 17.99, *p* < 0.001, partial *η*^2^ = 0.38. In contrast, in the emotional evaluation task, no significant difference was found in reaction times between moral (*M* = 575.62, *SD* = 77.84) and immoral words (*M* = 581.71, *SD* = 82.54), *F*(1,29) = 0.55, *p* = 0.464, partial *η*^2^ = 0.02. These findings only support the hypothesis that individuals respond more slowly to immoral words than to moral words in the moral evaluation task but do not support the hypothesis that individuals respond more quickly to immoral words than to moral words in the emotional evaluation task, thus failing to validate the emotional priority hypothesis. Interestingly, participants responded relatively faster to immoral words in the emotional evaluation task (*M* = 581.72, *SD* = 82.54) compared to the moral evaluation task (*M* = 598.00, *SD* = 93.68), although this difference was not statistically significant (*F*(1,29) = 2.79, *p* = 0.106, partial *η*^2^ = 0.09). This trend partially supports the emotional priority hypothesis, which posits that immoral words should be processed more quickly in emotional tasks and more slowly in moral evaluation tasks. In other words, although the hypothesis is not fully supported, the relatively faster responses to immoral words in emotional tasks align with the concept of emotional priority processing.

### 4.2. The Effect of Emotion and Reason

**Figure 4:**
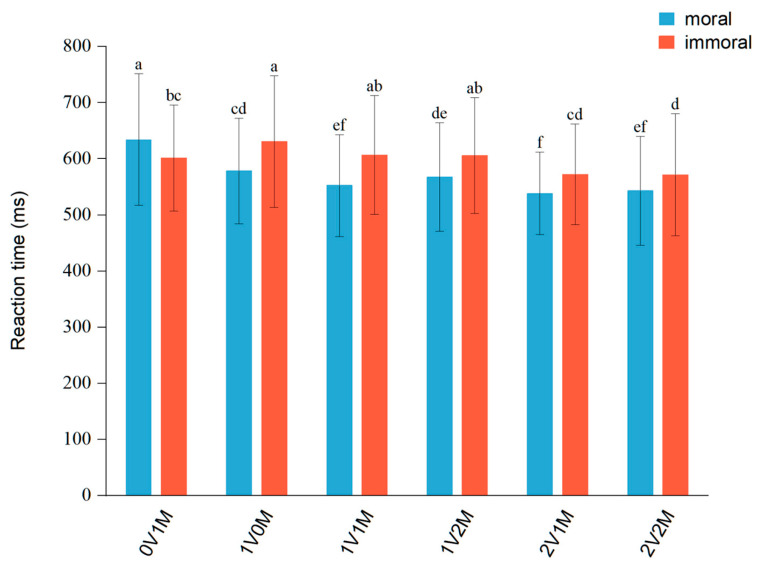
Results of reaction time for the moral evaluation task in different word groups. Note. Different letter marks indicate significant difference (*α* = 0.05). Error bars indicate the standard deviation. 0V1M indicates a category with low emotional and medium moral valence, 2V2M indicates a category with high emotional and high moral valence, and other symbols have the same naming rules.

Response times for each of the 12 moral vocabulary groups were analyzed using paired samples *t*-tests with FDR correction for multiple comparisons. Under the 1V0M category, individuals responded significantly slower to immoral words (*M* = 631.04, *SD* = 117.20) than to moral words (*M* = 578.36, *SD* = 94.03), *t*(29) = 4.49, corrected *p* < 0.001. Additionally, the other four categories showed faster response times for moral than immoral words (see Figure 4), consistent with the negative bias hypothesis towards immoral words. However, under the 0V1M category, individuals responded significantly faster to immoral words (*M* = 601.41, *SD* = 94.27) compared to moral words (*M* = 634.30, *SD* = 117.41), *t*(29) = −2.48, corrected *p* = 0.034. These conflicting results between categories suggest that differences in emotional or moral valence significantly influence reaction times. We then used a functional separation method to investigate the effect of two factors: assessing the impact of emotional valence by holding moral valence constant (0V1M vs. 2V1M) and assessing the impact of moral valence by holding emotional valence constant (1V0M vs. 1V2M).

Further analysis (see Figure 4) revealed that in immoral evaluation, the reaction time for immoral words in 0V1M (*M* = 601.41, *SD* = 94.27) was significantly slower compared to 2V1M (*M* = 572.25, *SD* = 89.77), *t*(29) = 2.27, corrected *p* = 0.050, and that the reaction time for immoral words in 1V0M (*M* = 631.04, *SD* = 117.20) was not significantly different from that in 1V2M (*M* = 605.78, *SD* = 103.55), *t*(29) = 1.86, corrected *p* = 0.105. These results suggest that immoral evaluation is only significantly affected by emotional valence. Similarly, in moral evaluation, the reaction time for moral words in 0V1M (*M* = 634.30, *SD* = 117.41) was significantly slower than in 2V1M (*M* = 538.47, *SD* = 73.81), *t*(29) = 6.73, corrected *p* < 0.001. There was no significant difference between 1V0M (*M* = 578.36, *SD* = 94.03) and 1V2M (*M* = 567.45, *SD* = 96.50), *t*(29) = 0.94, corrected *p* = 0.449. These results indicate that emotion and reason factors have different effects in moral evaluation, with individuals being significantly influenced by emotional valence but not by moral valence in moral and immoral evaluations. The findings support the emotional dominance hypothesis in moral evaluation. Additionally, in the 0V1M category, moral evaluation was slightly slower than immoral evaluation; however, in other categories, moral evaluation was faster than immoral evaluation. This contradictory finding suggests the presence of underlying psychological mechanisms. To validate the emotional dominance hypothesis and explore its underlying cognitive mechanisms, we then used the drift-diffusion model (DDM) to analyze the reaction time distribution.

### 4.3. The Underlying Mechanism of Emotion and Reason

The drift-diffusion model was used to analyze the reaction time distribution in the moral evaluation task. The upper threshold (a) of the model was fixed as the moral evaluation and the lower threshold as the immoral evaluation, with moral words having a positive drift rate (v) providing evidence for the moral evaluation and immoral words having a negative drift rate (v) providing evidence for the immoral evaluation. The model parameters (see Appendix B Table A2) for this study are as follows: twelve groups, each with its own set of drift rates (v), upper thresholds (a), and non-decision times (τ), all sharing a common starting point (z). These parameters reflect differences in evidence accumulation speed, decision caution, and information encoding time for the moral evaluation of vocabulary with different emotional and moral valences. Among them, the common starting point (z) reflects the prior bias towards either moral or immoral words.

**Figure A1:**
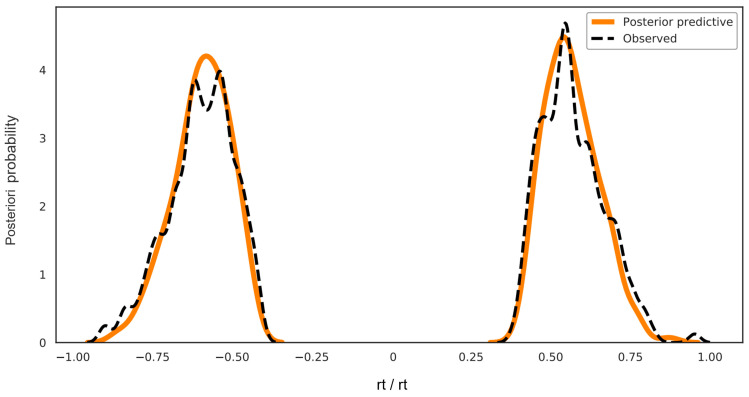
Posterior predictive checks (PPCs) for the bias model.

DDM fitting applies the HDDM (hierarchical Bayesian drift-diffusion model) Python package (version 0.8.0) [43,44]. Specifically, the package uses group-level priors to constrain parameter fitting at the individual level and is shown to improve the estimation accuracy in smaller samples [44]. Model fitting drew 60,000 samples from the posterior distribution, discarding the first 5000 “burn-in” samples. Trace and autocorrelation plots were examined to evaluate convergence. The estimated parameters were used to generate simulated data (posterior prediction checks) to assess model quality (see Appendix B Table A3, Figure A1). Given its hierarchical structure (individual and group levels) and Bayesian estimation, our statistical inference is based on the posterior distribution of the parameters at the group level. Bayesian inference testing is accomplished by comparing one posterior probability distribution to another (parametric posterior distribution in different word groups), resulting in a difference distribution compared to zero [45]. The mean of the difference distribution and the percentages of this distribution that are larger than zero and smaller than zero are reported [46]. For example, 95% < 0 < 5% means that 95% of the posteriors are smaller than 0, while 5% are larger than 0. Significance is considered when at least 95% of the distribution falls to the left or right of zero [47].

**Figure 5:**
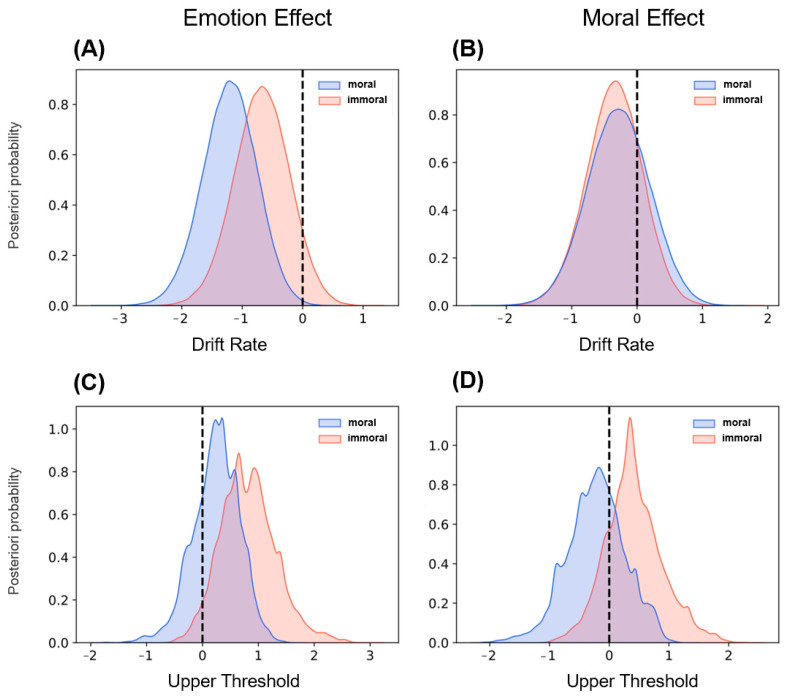
The difference distribution of the posterior probability of drift rate (v) and upper threshold (a) in different emotional (0V1M vs. 2V1M) and moral (1V0M vs. 1V2M) valence categories. (**A**) Drift rate (v) differences distribution in different emotional valence categories. (**B**) Drift rate (v) differences distribution in different moral valence categories. (**C**) Upper threshold (a) differences distribution in different emotional valence categories. (**D**) Upper threshold (a) differences distribution in different moral valence categories.

**Figure 6:**
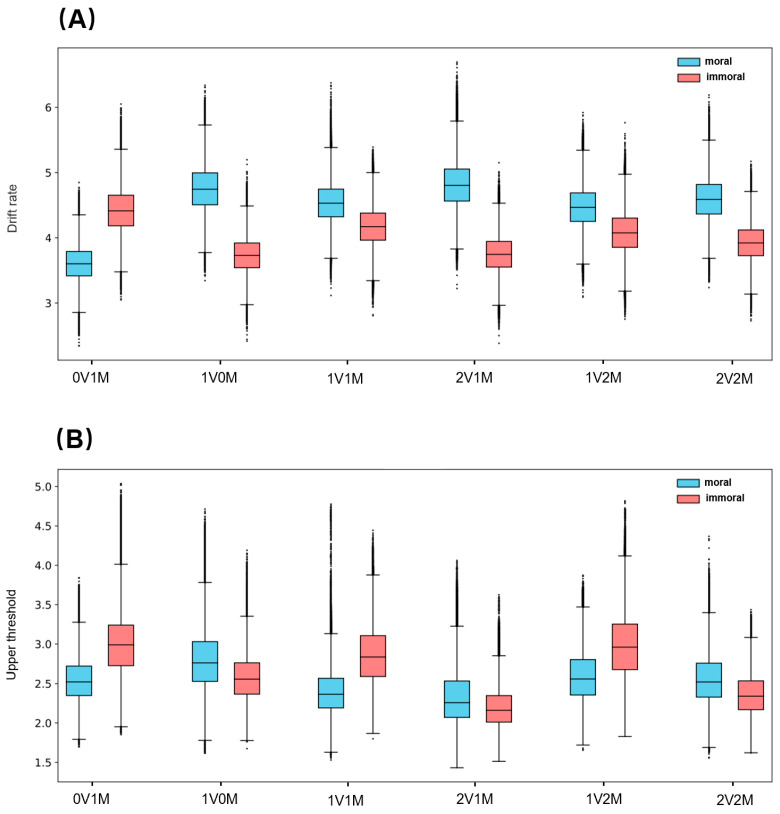
Posterior probability distribution in all group means. (**A**) Drift rate (v); (**B**) Upper threshold (a). Note. 0V1M represents a category with low emotional and medium moral valence, 2V2M indicates a category with high emotional and high moral valence, and other symbols follow the same naming conventions. The immoral drift rate shown in the figure is derived by taking the absolute value of the negative drift rate. The sign of the drift rate is used solely to indicate different evaluation choices.

HDDM fitting results showed that the drift rate (v) for immoral evaluation in 0V1M was significantly faster than that for moral evaluation (*M* = 0.81, 3.4% < 0 < 96.6%), explaining the faster evaluation of immoral words in this category. Moreover, the difference in drift rate (v) (see Figure 5A) between 2V1M and 0V1M for immoral evaluation was not significant (*M* = 0.67, 6.9% < 0 < 93.1%), whereas for moral evaluation, the drift rate (v) was significantly faster in 2V1M than in 0V1M (*M* = 1.21, 0.2% < 0 < 99.8%). This indicates that with increasing emotional valence, the difference in the evidence accumulation speed for immoral evaluation is insignificant, whereas it increases significantly for moral evaluation. This result clearly explains why moral evaluation is slower than immoral evaluation in the low-emotional category and faster in the high-emotional category. Consistent with the previous *t*-test, moral valence (1V0M vs. 1V2M) did not significantly affect drift rates (v) for either moral (*M* = −0.28, 72.1% < 0 < 27.9%) or immoral evaluation (*M* = −0.32, 79.3% < 0 < 20.7%, see Figure 5B). Furthermore, in immoral evaluation, higher emotional valence (2V1M vs. 0V1M) significantly lowered the upper threshold (a) (*M* = −0.64, 96.1% < 0 < 3.9%), while higher moral valence (1V2M vs. 1V0M) had no significant effect on the upper threshold (*M* = 0.34, 18.6% < 0 <81.4%). In moral evaluation, neither emotional valence (*M* = −0.34, 73.8% < 0 < 26.2%) nor moral valence (*M* = −0.17, 67.8% < 0 < 32.2%) significantly affected the upper threshold (a) (see Figure 5C,D), and the posterior distributions of parameters for other groups are shown in Figure 6. As shown in Figure 6A, the drift rate (v) of moral evaluations exhibits an overall increasing trend with higher emotional valence (0V1M < 1V1M < 2V1M), indicating a gradual increase in the speed of evidence accumulation. In contrast, the influence of moral valence is not significant (1V0M vs. 1V1M vs. 1V2M), with no significant difference observed between 1V0M and 1V2M (*M* = −0.28, 72.1% < 0 < 27.9%). As illustrated in Figure 6B, the decision threshold (a) for immoral evaluations shows an overall decreasing trend as emotional valence increases (0V1M < 1V1M < 2V1M), suggesting that the amount of evidence required to make an evaluation becomes progressively smaller. Similarly, the effect of moral valence remains insignificant (1V0M vs. 1V1M vs. 1V2M), with no significant difference between 1V0M and 1V2M (81.4% < 0 < 18.6%).

Combining the *t*-test and HDDM, the following conclusions can be drawn: For immoral evaluation, neither emotional nor moral valence significantly affects the evidence accumulation speed, but emotional valence significantly affects the degree of caution. For moral evaluation, emotional valence significantly affects the evidence accumulation speed, while neither emotional nor moral valence significantly affects the degree of caution. These results suggest that emotion and reason mechanisms differ in immoral and moral evaluations in moral lexical context. Emotional valence affects the degree of decision caution and the speed of evidence accumulation in the moral evaluation process, while moral valence has no significant impact on any psychological component of either evaluation type during the evaluation stage.

In addition, we verified the negative bias effect of immoral words through model comparison. This study found that the HDDM with negative bias information (DIC = −6521.129) outperformed the model without prior bias information (DIC = −6522.137). These results support the previous hypothesis [28] that immoral words are influenced by negative bias in the moral evaluation task. Since no differences exist in other parameters between the two models (see Appendix B Table A2), the slower reaction time to immoral words can be attributed partly to individuals’ negative bias towards immoral words, but primarily to the emotional valence of immoral words on decision caution and evidence accumulation speed.

## 5. Implications and Conclusions

This research applied a computational decision-making model to validate the applicability of the social intuitionist model in moral evaluation. We analyzed the mechanism of emotional and moral factors, respectively, to test the emotional dominance hypothesis and used different valence evaluation tasks to study the emotional priority hypothesis. Our findings support the emotional dominance hypothesis and introduce a new emotional mechanism into moral evaluation, providing methodological references and theoretical additions to a comprehensive theory of moral judgment.

The social intuitionist model, which posits that emotion determines moral judgment, has been increasingly challenged in recent years. Researchers have identified unreliable results for the hypothesis of emotional dominance and emotional priority within various research paradigms and moral scenarios [13,22]. At the same time, these researchers have a strong habit of studying immoral judgments [8,48], and the lack of attention to moral judgment situations makes the model limiting in generalization. Such issues necessitate further verification of the hypothesis when applying the model to moral evaluation. In response, our study applies the computational decision-making model (DDM) within a moral evaluation framework to conduct a quantitative analysis of moral and immoral evaluation behaviors. We found that emotional factors significantly affect moral evaluation, with different impacts observed in immoral versus moral evaluation. Specifically, words associated with higher negative emotions elicit more impulsive immoral evaluation, while those with higher positive emotions lead to quicker moral evaluation. These results are consistent with fMRI evidence that negative emotions impair cognitive control by taxing inhibitory regions of the prefrontal cortex, resulting in impulsive decisions [49]. Positive emotions, on the other hand, moderate the processing speed of working memory tasks by recruiting the fronto-parietal control network more frequently [50], thereby reducing its reaction time. Furthermore, experiments involving various valence evaluation tasks indicate a possible predisposition among individuals to prioritize emotional processing in moral evaluation. Our research supports the extension of the social intuitionist model’s emotional dominance hypothesis to moral evaluation and provides partial evidence for the moral priority hypothesis. In conclusion, we overcome the limitation of previous studies on the moral intuitionist model and provide concrete evidence supporting the effectiveness and applicability of the social intuitionist model, filling the gap on the types of moral evaluation in the moral judgment model.

These results enrich the understanding of moral practice. On one hand, heightened emotional arousal leads individuals to exhibit more risk-taking in immoral evaluations. In the fast-paced environment of social media, users often make judgments while emotionally charged, which can result in extreme viewpoints due to a lack of comprehensive information, neglecting the complexity of events. This phenomenon fosters “groupthink”, making users more susceptible to the emotional influences of others, thereby forming consensus-driven and emotional moral evaluations [51]. Furthermore, when confronted with immoral events, a strong sense of injustice can provoke “moral outrage”, compelling individuals to adopt extreme positions and criticize others morally [52]. This not only impacts individual moral evaluations but can also create significant public pressure within groups, influencing the overall societal moral assessment of certain events.

On the other hand, heightened emotional arousal can accelerate the speed of moral evaluations. This finding suggests that cultivating empathy may enhance individuals’ sensitivity to moral norms. By fostering empathy (emotional sharing, empathic concern, and perspective taking), individuals can better understand the feelings of others, enabling them to make quicker and more accurate judgments in moral dilemmas [53]. This also reduces impulsivity and bias in decision-making, thereby improving the moral quality of social interactions.

## 6. Limitations

A limitation of this article is that conclusions drawn solely from moral evaluation cannot represent other types of moral judgment. Despite this, existing evidence suggests that initial moral evaluation impacts following moral judgments. The culpable control model claims that initial emotional reactions to events influence final moral judgments [54]. The moral pervasiveness model posits that the initial evaluation of an event affects the entire information processing of moral judgment [55]. Therefore, the emotional impact at the evaluation stage also plays an important role in the real moral situation. Additionally, the results of the valence evaluation tasks did not fully support the emotion priority hypothesis, although the modeling results confirmed a negative bias towards immoral words in the process of moral evaluation. The moral pop-out effect may explain why there is no significant speed difference between immoral and moral words [56]. This effect suggests that moral vocabulary attract more attention and are recognized 100 ms faster than non-moral vocabulary. Given the already rapid pace of moral evaluation, the advantage conferred by the negative bias in moral vocabulary does not stand out in comparison to non-moral vocabulary, leading to an inconsistent result. Another limitation is this study’s limited ecological validity, which means we need to be careful when generalizing the findings. Part of this poor ecological validity comes from cultural differences in moral stimuli and judgment processes [57]. Additionally, the small sample size and narrow age range of participants limit the conclusions we can draw. Therefore, when applying these conclusions, we should carefully consider cultural differences and the diversity of the sample.

## 7. Future Research Directions

Our research only partially supports the emotional priority hypothesis in the social intuitionist model. Future investigations could explore experimental paradigms that can distinguish between reasoning and emotional processing in moral evaluation [58]. Moreover, due to the small sample size and the singularity of data types in this study, the conclusion that emotion dominates moral evaluation requires further validation with larger and more diverse samples. Therefore, future research could expand the sample population and utilize more tools, such as eye tracking, EEG, and fMRI, to provide supplemental evidence [18,27,59]. Researchers could develop computational models correlating neural and eye movement signals with DDMs [31,47] to explore emotional and reasoning processes in moral evaluation from a multimodal data and computational modeling perspective.

Given that this experiment only verifies the theory and lacks research in practical contexts, researchers can also refine moral judgment models applicable to real-life scenarios based on these findings. For instance, we can investigate whether high emotional arousal from moral events leads to riskier decisions during immoral evaluations. Conversely, we can explore whether moral evaluation is influenced solely by the degree of emotional arousal without changes in decision caution. Additionally, since moral evaluation is merely the first stage in the moral judgment process, it is important to understand how these different evaluation mechanisms influence subsequent behaviors. Finally, a comprehensive theory of moral judgment needs to be built on a clear foundation of knowledge, making it necessary to conduct more in-depth studies on this type of moral evaluation. Further research themes might investigate the elements contributing to differences in emotional mechanisms between immoral and moral evaluations.

## Figures and Tables

**Table 1:** Different groups of moral vocabulary.

			Moral Words			Immoral Words	
Emotional Valence		Low	Medium	High	Low	Medium	High
Moral valence	Low		1V0M			1V0M	
	Medium	0V1M	1V1M	2V1M	0V1M	1V1M	2V1M
	High		1V2M	2V2M		1V2M	2V2M

Note. Low, medium, and high emotional valence levels are represented by 0V to 2V; similarly, low, medium, and high moral valence levels are represented by 0M to 2M. For example, 2V2M represents a word category with high emotional valence and high moral valence.

## Data Availability

The data and code are publicly available and can be found at https://osf.io/7rb69/ (accesssed on 28 September 2024).

## Appendix A

**Table A1:** Experimental materials.

Category	Moral Word	Moral Valence	Emotional Valence	Immoral Word	Moral Valence	Emotional Valence
Low-emotional and medium-moral	抚育	7.26	6.74	禽畜	1.97	3.03
	俭朴	7.39	7.03	败坏	2.19	2.90
	豪爽	7.48	7.06	嘲讽	2.32	2.97
	谦虚	7.48	7.00	狰狞	2.48	3.03
	无私	7.45	7.03	哄骗	2.42	2.42
	节约	7.65	6.94	苛责	2.81	3.03
	安抚	7.45	7.00	奢靡	2.74	3.13
	解救	7.74	7.03	捉弄	2.61	2.90
Medium-emotional and low-moral	帮扶	7.10	6.84	刁钻	2.68	2.13
	分享	6.65	7.10	呵斥	3.52	2.58
	创新	7.19	6.97	偏袒	2.87	2.90
	保卫	7.19	7.10	阻碍	3.35	2.90
	援救	7.23	7.10	包庇	3.26	2.77
	温顺	6.52	7.32	阴谋	2.35	2.55
	安慰	7.03	7.19	荒唐	3.10	3.00
	保障	7.26	7.03	漠视	2.39	2.77
Medium-emotional and medium-moral	亲切	7.45	7.61	蛮横	2.48	2.29
	关怀	7.61	7.81	虚假	2.23	2.35
	孝敬	7.90	7.65	邪恶	2.16	2.19
	友好	7.81	7.74	抄袭	1.94	2.13
	信用	7.77	8.00	欺压	2.23	2.19
	团结	8.00	7.55	贪婪	2.52	2.71
	慷慨	7.81	7.45	诅咒	2.23	2.16
	助人	7.77	7.61	掠夺	2.16	2.45
Medium-emotional and High-moral	刚正	7.77	7.42	丑恶	1.87	2.52
	慈善	7.77	7.58	伪善	1.94	2.52
	尽责	7.81	7.48	抢占	1.97	2.48
	友爱	7.84	7.45	离间	2.06	2.23
	信义	7.90	7.39	凶横	2.10	2.26
	美德	7.97	7.39	挟持	2.26	2.58
	清廉	8.06	7.52	违约	1.97	2.58
	清正	8.16	7.55	抢劫	1.52	2.23
High-emotional and Medium-moral	光荣	7.74	8.23	欺负	2.55	2.16
	厚道	7.74	8.19	欺骗	2.10	1.94
	明智	7.48	7.87	不公	2.13	1.97
	善待	7.65	7.81	虚伪	2.10	1.90
	磊落	7.65	7.90	卑劣	2.10	1.97
	正直	7.68	7.94	卑鄙	2.13	2.00
	大方	7.55	7.71	腐朽	2.13	1.97
	亲和	7.68	7.71	专横	2.45	2.03
High-emotional and high-moral	尊重	7.97	7.81	残害	1.48	2.06
	诚实	8.32	7.87	陷害	1.87	2.06
	真挚	8.00	8.16	谋害	1.58	2.00
	公正	8.23	7.94	恐吓	1.81	2.10
	真诚	8.26	7.74	下流	1.58	1.97
	忠诚	8.16	7.97	腐败	1.52	1.90
	守信	8.10	8.09	阴险	1.81	2.06
	诚恳	8.06	7.90	遗弃	2.00	2.10

Note. Moral valence (1 = extremely immoral, 5 = neutral, 9 = extremely moral) and emotional valence (1 = extremely negative, 5 = neutral, 9 = extremely positive) was evaluated on a 1–9 scale via a questionnaire.

We collected data on word frequency, strokes (equivalent to the length of an English word, representing the complexity), emotional valence, and moral valence for the vocabulary. Since the scale ranges from 1 to 9, with 5 being neutral, we transformed the scores to make emotional and moral valence comparable in terms of intensity. Independent samples *t*-tests were conducted separately for moral and immoral words within each category. There were no significant differences in moral intensity, emotional intensity, strokes, and word frequency between moral and immoral words in the corresponding category, *p*s > 0.066. Word frequency information was obtained from an open dictionary database [60].

## Appendix B

**Table A2:** Posterior probability distribution of parameters in drift-diffusion model.

Model	Response	Category	Drift Rate (v)	Upper Threshold (a)	Non-Decision Times (τ)	Starting Point (z)
Bias Model	Moral evaluation	0V1M	3.60 [3.07, 4.16]	2.53 [2.01, 3.18]	0.26 [0.20, 0.29]	0.48 [0.44, 0.54]
		1V0M	4.73 [4.07, 5.48]	2.61 [2.20, 3.88]	0.26 [0.21, 0.31]	
		1V1M	4.51 [3.91, 5.16]	2.25 [1.88, 2.96]	0.27 [0.23, 0.30]	
		2V1M	4.79 [4.11, 5.53]	2.17 [1.69, 2.95]	0.29 [0.24, 0.32]	
		1V2M	4.44 [3.85, 5.12]	2.38 [1.96, 3.30]	0.26 [0.21, 0.29]	
		2V2M	4.59 [3.94, 5.25]	2.39 [1.69, 3.27]	0.25 [0.20, 0.28]	
	Immoral evaluation	0V1M	−4.39 [−5.13, −3.74]	3.04 [2.20, 3.93]	0.26 [0.20, 0.31]	
		1V0M	−3.74 [−4.29, −3.19]	2.53 [2.06, 3.19]	0.26 [0.23, 0.31]	
		1V1M	−4.20 [−4.76, −3.57]	2.77 [2.25, 3.60]	0.26 [0.21, 0.30]	
		2V1M	−3.76 [−4.32, −3.19]	2.13 [1.76, 2.70]	0.27 [0.23, 0.31]	
		1V2M	−4.04 [−4.74, −3.45]	3.10 [2.24, 3.81]	0.23 [0.17, 0.28]	
		2V2M	−3.92 [−4.49, −3.36]	2.26 [1.87, 2.91]	0.26 [0.22, 0.29]	
Non−bias Model	Moral evaluation	0V1M	3.54 [3.03, 4.10]	2.45 [2.06, 3.09]	0.26 [0.21, 0.30]	0.5
		1V0M	4.72 [4.04, 5.43]	2.84 [2.15, 3.58]	0.26 [0.22, 0.31]	
		1V1M	4.53 [3.89, 5.20]	2.38 [1.93, 3.15]	0.27 [0.22, 0.31]	
		2V1M	4.77 [4.10, 5.60]	2.31 [1.71, 3.17]	0.29 [0.23, 0.30]	
		1V2M	4.40 [3.80, 5.05]	2.56 [2.03, 3.14]	0.23 [0.17, 0.28]	
		2V2M	4.57 [3.95, 5.28]	2.25 [1.87, 2.83]	0.25 [0.20, 0.29]	
	Immoral evaluation	0V1M	−4.44 [−5.09, −3.77]	2.86 [2.26, 3.73]	0.26 [0.21, 0.31]	
		1V0M	−3.73 [−4.32, −3.21]	2.43 [2.02, 3.11]	0.27 [0.23, 0.31]	
		1V1M	−4.13 [−4.75, −3.55]	2.70 [2.14, 3.40]	0.26 [0.21, 0.30]	
		2V1M	−3.75 [−4.30, −3.22]	2.11 [1.74, 2.59]	0.28 [0.23, 0.30]	
		1V2M	−4.07 [−4.77, −3.44]	2.88 [2.24, 3.89]	0.26 [0.22, 0.29]	
		2V2M	−3.93 [−4.52, −3.38]	2.47 [2.01, 3.30]	0.26 [0.22, 0.29]	

Note. The 95% Bayesian credible intervals is indicated in brackets.

To ensure that the Markov chains all converged, we performed a visual inspection of the Markov chains with Gelman–Rubin statistics ($\hat{R}$ < 1.05) and a sufficient valid sample size (ESS > 800). To validate the bias model, we performed PPC (posterior predictive check) for the results (Table A3, Figure A1). For methods related to model parameter validation, please refer to this literature [61].

**Table A3:** Posterior predictive checks (PPCs) for decision outcomes (RT and choice).

	Observed	Mean	Std	SEM	MSE	Credible	Quantile	Mahalanobis
choice	0.501	0.500	0.499	0.000	0.249	TRUE	50.000	0.002
mean_moral	0.568	0.568	0.105	0.000	0.011	TRUE	56.580	0.002
std_moral	0.175	0.124	0.065	0.003	0.007	TRUE	83.653	0.786
10q_moral	0.401	0.440	0.072	0.002	0.007	TRUE	29.197	0.543
30q_moral	0.469	0.493	0.083	0.001	0.007	TRUE	42.276	0.289
50q_moral	0.529	0.544	0.098	0.000	0.010	TRUE	49.116	0.161
70q_moral	0.601	0.608	0.122	0.000	0.015	TRUE	54.577	0.059
90q_moral	0.781	0.720	0.175	0.004	0.034	TRUE	71.302	0.352
mean_immoral	−0.599	−0.596	0.110	0.000	0.012	TRUE	43.161	0.024
std_immoral	0.193	0.144	0.072	0.002	0.008	TRUE	80.680	0.678
10q_immoral	0.413	0.450	0.070	0.001	0.006	TRUE	30.708	0.523
30q_immoral	0.485	0.509	0.083	0.001	0.007	TRUE	41.856	0.288
50q_immoral	0.550	0.568	0.101	0.000	0.011	TRUE	47.801	0.174
70q_immoral	0.639	0.642	0.129	0.000	0.017	TRUE	55.668	0.022
90q_immoral	0.867	0.773	0.191	0.009	0.045	TRUE	75.007	0.494

Note. The table shows the fitting indexes of 500 reaction time data sets simulated by the bias model compared to experimental reaction time data. Observed indicates experimental data. Mean, Std, and SEM, respectively, indicate the mean, standard deviation, and standard error of the mean for the predicted data. MSE indicates the average squared prediction error. Credible indicates whether the observed data fall within the 95% credible interval of the simulated data. Quantile indicates the percentile position of the observed data within the distribution of simulated data. Mahalanobis indicates the distance between the observed data and the predicted data.

Using model parameter simulation to generate 500 datasets, all indicators show that the observed data and the simulated data match well, and the fit is generally good.
